# Optimal Treatment Strategy for a Tumor Model under Immune Suppression

**DOI:** 10.1155/2014/206287

**Published:** 2014-07-23

**Authors:** Kwang Su Kim, Giphil Cho, Il Hyo Jung

**Affiliations:** Department of Mathematics, Pusan National University, Busan 609-735, Republic of Korea

## Abstract

We propose a mathematical model describing tumor-immune interactions under immune suppression. These days evidences indicate that the immune suppression related to cancer contributes to its progression. The mathematical model for tumor-immune interactions would provide a new methodology for more sophisticated treatment options of cancer. To do this we have developed a system of 11 ordinary differential equations including the movement, interaction, and activation of NK cells, CD8^+^T-cells, CD4^+^T cells, regulatory T cells, and dendritic cells under the presence of tumor and cytokines and the immune interactions. In addition, we apply two control therapies, immunotherapy and chemotherapy to the model in order to control growth of tumor. Using optimal control theory and numerical simulations, we obtain appropriate treatment strategies according to the ratio of the cost for two therapies, which suggest an optimal timing of each administration for the two types of models, without and with immunosuppressive effects. These results mean that the immune suppression can have an influence on treatment strategies for cancer.

## 1. Introduction

Cancer is a leading cause of death worldwide. Cancer is a term used for diseases in which abnormal cells divide without control and are able to invade some tissues. The possibility that cancers can be eradicated by specific immune responses has been the impetus for the field of tumor immunology. The existence of immune surveillance, which was proposed by Macfarlane Burnet in the 1950s, has been demonstrated by the incidence of some types of tumors in immunocompromised experimental animals and humans [1].

Chemotherapy and immunotherapy are typical treatment methods of cancer. Chemotherapy directly targets the transformed tumor cell. But chemotherapy has some general side effects such as hair loss, a sore mouth, vomiting, and diarrhea [2]. Immunotherapy is treatment method that uses body's own immune system to help fighting cancer. There are such many treatment methods for cancer. It is important to know how to combine these treatment methods as well as to find treatment methods for cancer treatment. Although the immune system is very complex, we may suggest more effective treatment strategies for cancer control through mathematical models associated with the immune system. So, identifying a mathematical model of tumor-immune interactions that mediate the immune responses and immune suppression would provide a new strategy for more sophisticated treatment methods.

There are amount of papers that deal with mathematical models about tumor-immune interaction. In Kuznetsov et al. [3], a mathematical model describing the cytotoxic T lymphocyte response to the growth of an immunogenic tumor was proposed. Kirschner and Panetta [4] presented a mathematical model describing the dynamics between tumor cells, immune-effector cells, and IL-2. They explained both short-tumor oscillations in tumor sizes and long-term tumor relapse. In de Pillis et al. [5], they proposed a mathematical model which was based on de Pillis and Radunskaya's model [6] and includes tumor cells and three immune cells as well as two drug concentrations in the bloodstream and the model described bifurcation-like behavior to be reproduced under particular realistic conditions. In addition, they showed that combination therapy has more effects than only one therapy for tumor control through numerical simulations. The model in [7] extended de Pillis' model [6] and identified appropriate values for the parameters of the new model according to recent empirical data. de Pillis' paper [8] dealt with optimal control problem for the model of tumor-immune interactions with chemotherapy. They characterized the optimal controls related to drug therapy, including a quadratic control, a linear control, and state constraint. Also, Engelhart et al. [9] introduced four different cancer mathematical models of chemotherapy from the literature and compared with results of optimal control on their models. Here we note that the above-mentioned all models describe tumor-immune interactions without immune suppression. Although the immune system response tumor cells, the fact that each year many people die from cancer suggests that immune response to tumor cells is often ineffective. In fact, an immunosuppressive effect is known to be one of the main causes of such phenomenon so that there are several mechanisms by which tumor cells appear to evade the immune system [1, 10]. The paper of Vaage [11] gave that the immune system plays a significant role in removing the tumors of cancer and pointed out that it does not always work. Chouaib et al. [12] found out that immune suppression obstructs the immune system in removing tumors. Recently, Robertson-Tessi et al. [13] proposed a mathematical model of tumor-immune interactions. They showed only existence of optimal antigenicity maximizing the immune response.

So even though considering immunosuppressive effects in the tumor model is important, mathematical modeling for tumor-immune interactions under immune suppression is still rare. Here we focus on finding optimal treatment strategies for tumor-immune models under immune suppression.

For this goal, combining the ideas of [7, 13], we consider an improved tumor dynamic model including three types of immune response: NK cell as innate immune response; CD8^+^T cell, CD4^+^T cell, and IL-2 as adaptive immune response; and regulatory T cell, TGF-*β*, and IL-10 as a role of immune suppression. Actually we have developed a system of 11 ordinary differential equations describing the movement, interaction, and activation of tumor-immune system under immune suppression. We set up a controlled tumor immune system incorporating two control measures: immunotherapy and chemotherapy. In addition, we establish the existence of an optimal control for the control system and also drive an optimal control and the optimality system using optimal control techniques. Using the numerical simulations, we suggest the optimal treatment strategies on the base of time for the control system under immunosuppressive effects. We find out that if the cost of chemotherapy is more expensive than that of immunotherapy, then the optimal treatment strategy for the model without immunosuppressive effects needs to be taken for a longer time for chemotherapy comparing immunotherapy. On the other hand, when the model has immunosuppressive effects and the cost of chemotherapy is more expensive than that of immunotherapy, the optimal treatment strategy for the model needs to be taken for a longer time for immunotherapy comparing chemotherapy.

The rest of this paper is organized as follows. In Section 2, we give the mathematical model for the tumor-immune system and describe the parameters used in the equations with optimal control problem. The necessary conditions for an optimal control and the corresponding states are derived using Pontryagin's maximum principle. In Section 3, we show that the efficacy of immunotherapy and chemotherapy using numerical simulation and the resulting optimality system and the parameter sensitivity analysis are numerically solved. Finally, discussion and conclusions are given in Section 4.

## 2. Mathematical Modeling and Optimal Control Problem for a Tumor-Immune System

**Figure 1:**
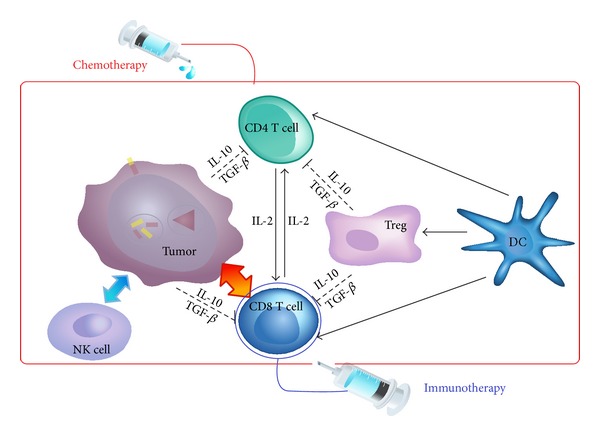
Tumor and immune response to chemotherapy and immunotherapy.

If a tumor cell formed in our body, firstly by innate immune response, NK cells kill the tumor cells. By the adaptive immune response, after dendritic cells knew about tumor cell's information, dendritic cells convey information about tumor to other T cells. CD4^+^T cells and CD8^+^T cells activated each other by cytokine IL-2. By influence of immune suppression, regulatory T cells and tumor cell limit functions of other T cells by cytokine IL-10 and TGF-*β*. Next, CD8^+^T cells directly kill tumor cells by adaptive immune response [1] (see Figure 1).

In this section, we describe the above-mentioned three important immune responses and they are represented in Figure 1. Our baseline model [13] describes adaptive immune response and influence of immune suppression, but they do not include effects of the innate immune response. Although de Pillis' model [7] includes the adaptive immune response and the innate immune response, they did not consider the immune suppression to their model. Motivated by these two papers, we make mathematical model describing tumor-immune interactions which include three immune responses, chemotherapy, and immunotherapy by using mathematical model.

This model is based on Robertson-Tessi's model [13]. Their model used 12 biological variables: nine cell types and three cytokines. All three types of T cells are specific to the tumor antigen and each of the three T-cell populations is broken into three subpopulations. The memory T cell (*M*_*E*_, *M*_*H*_, and *M*_*R*_) is a pool of T cell precursors, which are activated by dendritic cells. There is a short-lived activation phase (*A*_*E*_, *A*_*H*_, and *A*_*R*_) where T cell proliferation occurs. The fully functional T cell phase (*E*, *H*, *R*) consists of the cells that kill the tumor cells, produce cytokines, and suppress the immune response. The dendritic cell population has two subpopulations. The unlicensed state, *U*(*t*), is a mature dendritic cell that collects antigen from the tumor and then interacts with helper cells. After licensing, the dendritic cell, *D*(*t*), is free to interact with all T cells, causing them to activate. For update to our baseline model, firstly, we assume logistic model for growth of tumor. Since experimental data of tumor growth for the kinds of tumor is different, there are many growth curves to describe tumor growth. So we use more a simple logistic growth curve than the tumor growth curve in Robertson-Tessi's model [13]. Also, by substitute *T* for T* in [13], we exclude effects of spatial access to the tumor cells. In Robertson-Tessi's model [13], all three types of T cells are activated in the same basic way. The memory T cells enter a brief activation phase where proliferation is rapid, and then they become fully functional T cells. Thus, we may omit states of *A*_*E*_, *A*_*H*_, and *A*_*R*_ because the stages of *A*_*E*_, *A*_*H*_, and *A*_*R*_ only activate stages of *E*, *H*, and *R*, respectively.

Secondly, we add −*c*_3_ *E*_*T*_ *T* for describing CD8^+^T cell death by exhaustion of tumor-killing resources in the third equation of the system (1) [7]. Thirdly, we include the state of NK cells and describe innate immune response [7]. Finally, for describing death of cells due to chemotherapy, we include state of chemotherapy concentration and chemotherapy-induced cell death term [5]. The completed model is described by the following system of differential equations:

(1) dTdt=aT(1−bT)−c1TN −dTETET+eT1(1+g1(GT/ET))(1+(S/s1)) −kT(1−e−M)T,dNdt=b1−dNN−c2TN+pNNI2qN+I2−kN(1−e−M)N,dETdt=α1I2DLmE(1+(S/s2))(i1+I2)(d1+DL) −c3ETT−kET(1−e−M)ET+vE+ω1uE(t),dDUdt=pT(1+(I10/i2))(1+(GT/g2)) −γ1DU1+(DU/mH)−dDUDU−kDU(1−e−M)DU,dDLdt=γ1DU1+(DU/u1)−dDLDL,dHTdt=α2I2(DL+DU)mH(1+(S/s2))(i1+I2)(d1+DL+DU) −γ2HTSs3+S−dHTHT−kHT(1−e−M)HT,dGTdt=γ2HTSs3+S+α3I2DLmG(i1+I2)(d1+DL) −dGTGT−kGT(1−e−M)GT,dSdt=p1GT+p2T−dsS,dI2dt=α4HT(1+(I10/i3))(1+(S/s4))−dI2I2,dI10dt=p3GT+p4T−dI10I10,dMdt=−dMM+vM+ω2uM(t).

Here *T*(*t*) represents number of tumor cells. And immune system is partitioned into the innate immune response (*N*(*t*) NK cell numbers), adaptive immune response (*E*_*T*_(*t*) (CD8^+^T cell numbers), *D*_*U*_(*t*) (unlicensed dendritic cell numbers), *D*_*L*_ (licensed dendritic cell numbers), *H*_*T*_(*t*) (CD4^+^T cell numbers), *I*_2_(*t*) (IL-2)), and immune suppression (*G*_*T*_(*t*) (regulatory T cell numbers), *S*(*t*) (TGF-*β*), *I*_10_(*t*) (IL-10)).

When we inject chemotherapy, tumor cells are killed but other cells are damaged also. Among the many methods of immunotherapy, we use method injecting CD8^+^T cells directly. *M*(*t*) is chemotherapy drug concentration in the bloodstream. The parameters *v*_*E*_(*t*) and *v*_*M*_(*t*) represent amounts of chemotherapy and immunotherapy, respectively [7].

For the immunochemotherapy, we follow amount of dosage suggested by the manufacturers of the drug Adria. We use the upper end of the dosing range to arrive at *v*_*M*_ = 2.3869 mg/L per every 21 days for chemotherapy and the amount of injected CD8^+^T cells number is *v*_*L*_ = 1.77 × 10^10^. Since the units of parameters are different in two models [7, 13], we have converted ng/mL into IU/L by using the specific activity of IL-10 (3 × 10^6^ IU/mg) and TGF-*β* (2 × 10^7^ IU/mg). *i*_1_ = 0.3 ng/mL is taken from [13]. We arrive at our value for *i*_1_ by employing the specific activity of IL-2 ((1.8 × 10^7^)/1.1 IU/mg) to convert molar concentration to IU/L [7]. So we have

(2) $$\begin{array}{c} \frac{0.3\;\text{ng}}{1\;\text{mL}}\times \frac{1\;\text{mg}}{10^{6}\;\text{ng}}\times \frac{1000\;\text{mL}}{1\;\text{L}}\times \frac{1.8\times 10^{7}\;\text{IU}}{1.1\;\text{mg}}=4909\;\text{IU/L}. \end{array}$$

We summarized the model's term descriptions and value of parameters in Table 1.

In this work, we introduce two control functions *u*_*E*_(*t*) and *u*_*M*_(*t*) representing amounts of immunotherapy and chemotherapy, respectively. The parameters *ω*_1_ and *ω*_2_ are weight factors. We assume that number of injected CD^+^8 T cells for immunotherapy is 175000000 during 50 days. So we put the values of *ω*_1_ is 3500000. And we assume that amounts of chemotherapy are 25 during 50 days. So we put the values of *ω*_2_ to be 0.5.

Next we consider a cost functional as follows:

(3) $$\begin{array}{c} F\left(u_{E},u_{M}\right)=\overset{t_{f}}{\underset{0}{\int }}AT\left(t\right)+Bu_{E}{\left(t\right)}^{2}+Cu_{M}{\left(t\right)}^{2}dt. \end{array}$$

This functional includes the number of tumor cells, amounts of chemotherapy, and amounts of immunotherapy. In words, we are minimizing the number of tumor cells, amounts of chemotherapy, and amounts of immunotherapy. In the objective cost functional, the quantities *A*, *B*, and *C* represent the weight constants of tumor cell numbers, for immunotherapy and chemotherapy, respectively. The costs associated with immunotherapy and chemotherapy are described in the terms *Bu*_*E*_(*t*) and *Cu*_*M*_(*t*), respectively. Our goal is to minimize the cost functional (3), which is called the optimal control problem. That is, the optimal control problem is to seek optimal control functions (*u*_E_*(*t*), *u*_M_*(*t*)) such that

(4) $$\begin{array}{c} F\left(u_{E}^{*},u_{M}^{*}\right)=\min\left\{F\left(u_{E},u_{M}\right),\left(u_{E},u_{M}\right)\in U\right\} \end{array}$$

is subject to the system (1) and appropriate initial conditions are given at *t* = 0, where the control set is defined as

(5) U={u=(uE,uM) ∣ ui(t)  is  Lebesgue  measurable,0≤ui(t)≤1,  t∈[0,T]  for  i=E,M}.

Pontryagin's maximum principle is used to solve this optimal control problem and the derivation of the necessary conditions. First we prove the existence of an optimal control for problem (4) and then derive the optimality system.

**Theorem 1 .**

Given that the cost functional *F*(*u*_*E*_, *u*_*M*_) = ∫_0_^*t*_*f*_^ *AT*(*t*) + *Bu*_*E*_(*t*)^2^ + *Cu*_*M*_(*t*)^2^ *dt* and the control set *U* given by (5) is measurable, there exists on optimal control u* = (*u*_E_*, *u*_M_*) such that *F*(*u*_E_*, *u*_M_*) = min⁡{*F*(*u*_*E*_, *u*_*M*_), (*u*_*E*_, *u*_*M*_) ∈ *U*}.

**Proof**

To prove the existence of an optimal control, we use the result in [14]. Note that the control and the state variable are nonnegative values. In this minimizing problem, the necessary convexity of the objective functional in *u*_*E*_, *u*_*M*_ are satisfied. The set of all the control variables (*u*_*E*_, *u*_*M*_) ∈ *U* is also convex and closed by definition. The optimal system is bounded which determines the compactness needed for the existence of the optimal control. In addition, the integrand in functional (3), *AT*(*t*) + *Bu*_*E*_(*t*)^2^ + *Cu*_*M*_(*t*)^2^, is convex on the control set *U*. Also we can easily see that there exist a constant *δ* > 1 and numbers *ϕ*_1_, *ϕ*_2_ such that

(6) $$\begin{array}{c} F\left(u_{E},u_{M}\right)\geq \phi_{1}{\left(u_{E}^{2}+u_{M}^{2}\right)}^{\delta /2}-\phi_{2}, \end{array}$$

because the state variables are bounded, which completes the existence of an optimal control.

In order to find an optimal solution of the system, first we should find the Lagrangian and Hamiltonian for the optimal control problem (4). The Lagrangian of the control problem is given by

(7) $$\begin{array}{c} L=AT\left(t\right)+Bu_{E}{\left(t\right)}^{2}+Cu_{M}{\left(t\right)}^{2}. \end{array}$$

We seek for the minimal value of the Lagrangian. To do this, we define the Hamiltonian function *H* for the system, where *λ*_*i*_, *i* = 1,…, 11, are the adjoint variables:

(8) H=AT(t)+BuE(t)2+CuM(t)2 +λ1[aT(1−bT)−c1TN−dTETET+eT1(1+g1(GT/ET))(1+(S/s1))−kT(1−e−M)T] +λ2[b1−dNN−c2TN+pNNI2qN+I2−kN(1−e−M)N] +λ3[α1I2DLmE(1+(S/s2))(i1+I2)(d1+DL)−c3ETT−kET(1−e−M)ET+vE+ω1uE(t)] +λ4[pT(1+(I10/i2))(1+(GT/g2))−γ1DU1+(DU/u1)−dDUDU−kDU(1−e−M)DU] +λ5[γ1DU1+(DU/u1)−dDLDL] +λ6[α2I2(DL+DU)mH(1+(S/s2))(i1+I2)(d1+DL+DU)−γ2HTSs3+S−dHTHT−kHT(1−e−M)HT] +λ7[γ2HTSs3+S+α3I2DLmG(i1+I2)(d1+DL)−dGTGT−kGT(1−e−M)GT] +λ8[p1GT+p2T−dsS] +λ9[α4HT(1+(I10/i3))(1+(S/s4))−dI2I2] +λ10[p3GT+p4T−dI10I10] +λ11[−dMM+vM+ω2uM(t)].

In order to derive the necessary conditions, we use Pontryagain's maximum principle [15] as follows.

If (*x*, *u*) is an optimal solution of an optimal control problem, then there exists a nontrivial vector function *λ* = (*λ*_1_, *λ*_2_,…, *λ*_*n*_) satisfying the following inequalities:

(9) dxdt=∂H(t,x,u,λ)∂λ,0=∂H(t,x,u,λ)∂u,dλdt=−∂H(t,x,u,λ)∂x.

We now drive the necessary conditions that optimal control functions and corresponding states must satisfy. In the following theorem, we present the adjoint system and control characterization.

**Theorem 2 .**

Given an optimal control u* = (*u*_E_*, *u*_M_*) and a solution y* = (T*, N*, *E*_T_*, *D*_U_*, *D*_L_*, *H*_T_*,*G*_T_*, S*, *I*_2_*, *I*_10_*, M*) of the corresponding state system (1), there exists adjoint variables *λ*_*i*_, *i* = 1,…, 11, satisfying

(10) λ1′(t)=−∂H∂T=−A −λ1[a−2abT−c1N−dET2(1+g1(GT/ET))(1+(S/s1))(ET+eT)2−kT(1−e−M)] −λ2[−c2N]−λ3[−c3ET] −λ4[p(1+(I10/i2))(1+(GT/g2))] −λ8[p2]−λ10[p4],λ2′(t)=−∂H∂N=−λ1[−c1T] −λ2[−dN−c2T+pNI2qN+I2−kN(1−e−M)],λ3′(t)=−∂H∂ET=−λ1[−dT1+(S/s1)×(g1ET2G+eET2T+2eg1TGTET(ET+eT)2(ET+g1GT)2)] −λ3[−c3T−kET(1−e−M)],λ4′(t)=−∂H∂DU=−λ4[−γ1(1+(DU/u1))2−dDU−kDU(1−e−M)] −λ5[γ1(1+(DU/u1))2] −λ6[α2I2mHd1(1+(S/s2))(i1+I2)(d1+DL+DU)2],λ5′(t)=−∂H∂DL=−λ3[α1ImEd1(1+(S/s2))(i1+I2)(d1+DL)2] −λ5[−dDL] −λ6[α2ImHd1(1+(S/s2))(i1+I2)(d1+DL)2] −λ7[α3ImGd1(1+(S/s2))(i1+I2)(d1+DL)2],λ6′(t)=−∂H∂HT=−λ6[−γ2SS3+S−dHT−kHT(1−e−M)] −λ7[γ2SS3+S] −λ9[α4(1+(I10/i3))(1+(S/s4))],λ7′(t)=−∂H∂GT=−λ1[dTET2g1(ET+eT)(ET+g1GT)2(1+(S/s1))] −λ4[−pTg2(1+(I10/i2))(g2+GT)2] −λ7[−dGT−kGT(1−e−M)] −λ8[p1]−λ10[p3],λ8′(t)=−∂H∂S=−λ1[dTET2s1(ET+eT)(ET+g1GT)(s1+S)2] −λ3[α1I2DLmEs2(i1+I2)(d1+DL)(s2+S)2] −λ6[−α2I2(DL+DU)mHs2(i1+I2)(d1+DL+DU)(s2+S)2−γ2Hs3(s3+S)2] −λ7[γ2HTs3(s3+S)2]−λ8[−dS] −λ9[−α4HTs4(1+(I10/i3))(s4+S)2],λ9′(t)=−∂H∂I2=−λ2[pNNqN(qN+I2)2] −λ3[α1DLmEi1(1+(S/s2))(d1+DL)(i1+I2)2] −λ6[α2(DL+DU)mHi1(1+(S/s2))(d1+DL+DU)(i1+I2)2] −λ7[α3DLmGi1(d1+DL)(i1+I2)2]−λ9[−dI2],λ10′(t)=−∂H∂I10=−λ4[−pTi2(1+(GT/g2))(i2+I10)2] −λ9[−α4HTi3(1+(S/s4))(i3+I10)2]−λ10[−dI10],λ11′(t)=−∂H∂M=−λ1[−kTe−MT]−λ2[−kNe−MN] −λ3[−kETe−MET] −λ4[−kDUe−MDU]−λ6[−kHTe−MHT] −λ7[−kGTe−MGT]

with transversality conditions

(11) $$\begin{array}{c} \lambda_{i}\left(t_{end}\right)=0,\;i=1,2,\ldots ,11. \end{array}$$

Furthermore, the control functions *u*_E_*, *u*_M_* are given by

(12) $$\begin{array}{c} u_{E}^{*}=\min\left\{1,\;\max\left\{0,R_{E}\right\}\right\}\;where\;\;R_{E}=\frac{-\lambda_{3}\omega_{1}}{2B},\\ u_{M}^{*}=\min\left\{1,\;\max\left\{0,R_{M}\right\}\right\}\;where\;\;R_{M}=\frac{-\lambda_{11}\omega_{2}}{2C}. \end{array}$$

**Proof**

To determine the adjoint equations and the transversality conditions we use the Hamiltonian (8). The adjoint system results from Pontryagin's maximum principle [15] are as follows:

(13) $$\begin{array}{c} \lambda_{1}^{\prime }\left(t\right)=-\frac{\partial H}{\partial T},\lambda_{2}^{\prime }\left(t\right)=-\frac{\partial H}{\partial N},\ldots ,\lambda_{11}^{\prime }\left(t\right)=-\frac{\partial H}{\partial M} \end{array}$$

with *λ*_*i*_(*t*_*f*_) = 0.

To get the characterization of the optimal control given by (12), solving the equations,

(14) $$\begin{array}{c} \frac{\partial H}{\partial u_{E}}=0,\;\;\frac{\partial H}{\partial u_{M}}=0 \end{array}$$

on the interior of the control set and using the property of the control space *U*, we can derive the desired characterization (12).

Here we call formulas (12) for u* the characterization of the optimal control. The optimal control and the state are found by solving the optimality system, which consists of the system (1), the adjoint system (10), initial conditions at *t* = 0, boundary conditions (11), and the characterization of the optimal controls (12). To solve the optimality system, we use the initial and transversality conditions together with the characterization of the optimal control (*u*_E_*, *u*_M_*) given by (12). In addition, the second derivative of the Lagrangian with respect to *u*_*E*_, *u*_*M*_, respectively, are positive, which shows that the optimal problem is minimum at controls *u*_E_*, *u*_M_*. By substituting the values of *u*_E_*, *u*_M_* in the control system (1), we get the following system:

(15) dT∗dt=aT∗(1−bT∗)−c1T∗N∗−dT∗ET∗ET∗+eT∗1(1+g1(GT∗/ET∗))(1+(S∗/s1))−kT(1−e−M∗)T∗,dN∗dt=b1−dNN∗−c2T∗N∗+pNN∗I2∗qN+I2∗−kN(1−e−M∗)N∗,dET∗dt=α1I2∗DL∗mE(1+(S∗/s2))(i1+I2∗)(d1+DL∗)−c3ET∗T∗−kET(1−e−M∗)ET∗+min⁡{1,max⁡{0,RE}},dDU∗dt=pT∗(1+(I10∗/i2))(1+(GT∗/g2))−γ1DU∗1+(DU∗/u1)−dDU∗DU∗−kDU∗(1−e−M∗)DU∗,dDL∗dt=γ1DU∗1+(DU∗/u1)−dDLDL∗,dHT∗dt=α2I2∗(DL∗+DU∗)mH(1+(S∗/s2))(i1+I2∗)(d1+DL∗+DU∗)−γ2HT∗S∗s3+S∗−dHTHT∗−kHT(1−e−M∗)HT∗,dGT∗dt=γ2HT∗S∗s3+S∗+α3I2∗DL∗mG(i1+I2∗)(d1+DL∗)−dGTGT∗−kGT(1−e−M∗)GT∗,dS∗dt=p1GT∗+p2T∗−dsS∗,dI2∗dt=α4HT∗(1+(I10∗/i3))(1+(S∗/s4))−dI2I2∗,dI10∗dt=p3GT∗+p4T∗−dI10∗I10∗,dM∗dt=−dMM∗+min⁡{1,max⁡{0,RM}}

with H* at (*t*, T*, N*, *E*_T_*, *D*_U_*, *D*_L_*, *H*_T_*, *G*_T_*, S*, *I*_2_*, *I*_10_*, M*);

(16) H∗=AT∗+Bmin⁡{1,max⁡{0,RE}}∗2 +Cmin⁡{1,max⁡{0,RM}}∗2 +λ1dT∗dt+λ2dN∗dt+λ3dET∗dt +λ4dDU∗dt+λ5dDL∗dt+λ6dHT∗dt +λ7dGT∗dt+λ8dS∗dt+λ9dI2∗dt +λ10dI10∗dt+λ11dM∗dt.

To find out the optimal control and state, we will numerically solve the above system (15) and (16).

## 3. Numerical Simulations

In this section, we give the numerical results for the effects of chemotherapy and immunotherapy, and optimal control strategy on the tumor-immune model. In our simulations, we consider two initial tumor sizes but keep all other initial state values:

(17) $$\begin{array}{c} N\left(0\right)=2.5\times 10^{8},\;\;E_{T}\left(0\right)=5.268\times 10^{5},\\ D_{U}\left(0\right)=4.725\times 10^{7},\;\;D_{L}\left(0\right)=10,\\ H_{T}\left(0\right)=1.0536\times 10^{6},\;\;G_{T}\left(0\right)=1.795\times 10^{5},\\ I_{2}\left(0\right)=1173,\;\;S\left(0\right)=0,\\ I_{10}\left(0\right)=0,\;\;M\left(0\right)=0. \end{array}$$

Initial conditions of NK cells, CD8^+^T cells, and IL-2 are from [7]. We note that initial conditions of CD4^+^T cells and regulatory T cells are derived by initial condition of CD8^+^T cells. Since the typical ratio of CD8^+^T cells to CD4^+^T cells to regulatory T cells is approximately 3 : 6 : 1 [1], we have

(18) HT(0)=5.268×105×2=1.0536×106,GT(0)=5.268×105×13=1.795×105.

We assume initial conditions of *D*_*L*_, *S*, *I*_2_, and *I*_10_. Our normal dendritic cell counts are in agreement with those in the product insert of Miltenyi Biotec's blood dendritic cell enumeration kit, which tabulated a total mean dendritic cell count in normal volunteers of 2.8 × 10^7^ cells/L [16]. In [17], they report that melanoma patients have more circulating dendritic cell per milliliter of blood compared with normal controls. Their mean dendritic cell count for normal volunteers is 64 cells/mL of whole blood, whereas their melanoma patients had mean dendritic cell counts of 108 cells/mL for stage 4 diseases, respectively. So we calculated initial conditions of dendritic cells as follows:

(19) DU(0)=2.8×107  cells/L×108  cells/mL64  cells/mL=4.725×107  cells/L.

**Figure 2:**
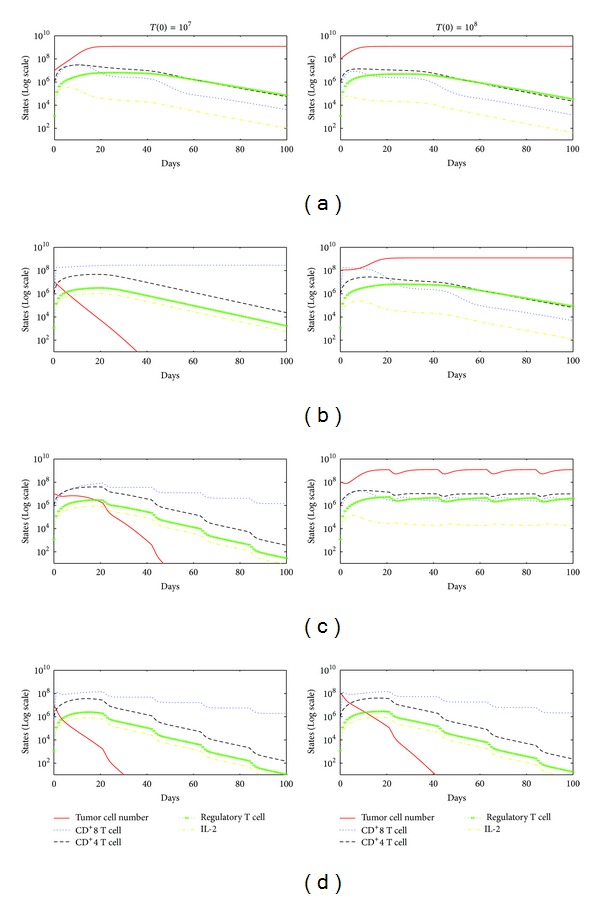
(a) No treatment, (b) immunotherapy, (c) chemotherapy, (d) immunotherapy, and chemotherapy.

In Figure 2(a), with no therapy, the immune system is not able to destroy the tumor cells with initial tumor size of *T*(0) = 10^8^ cells as well as *T*(0) = 10^7^ cells.  Figure 2(b) with immunotherapy and Figure 2(c) with chemotherapy show the results of the system (1). In both Figures 2(b) and 2(c), tumor cells are only decreased by the immune system when the initial tumor cell number *T*(0) = 10^7^.

Finally, Figure 2(d) displays the effects of combined therapy on initial tumor sizes *T*(0) = 10^7^ cells and *T*(0) = 10^8^ cells. In this case, the tumor is rapidly destroyed in two cases.

**Figure 3:**
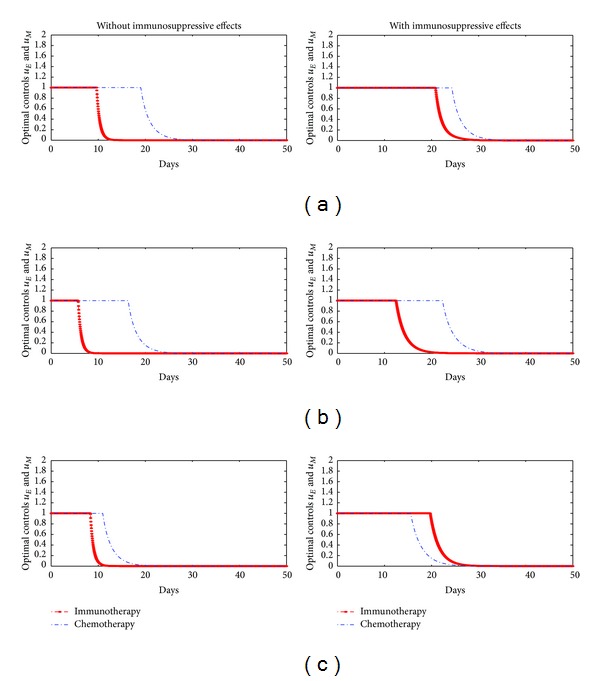
Optimal controls when initial value of tumor cells is 10^7^, (a) *A* = 1, *B* = 1, *C* = 1, (b) *A* = 1, *B* = 1000, *C* = 10, and (c) *A* = 1, *B* = 10, *C* = 1000.

**Figure 4:**
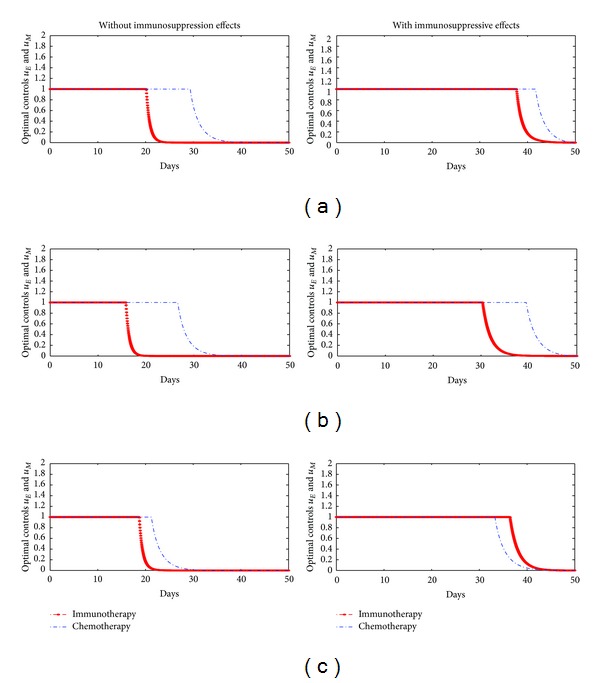
Optimal controls when initial value of tumor cells is 10^8^, (a) *A* = 1, *B* = 1, *C* = 1, (b) *A* = 1, *B* = 1000, *C* = 10, and (c) *A* = 1, *B* = 10, *C* = 1000.

For the case of exception immunosuppressive effects, we assume that initial values of regulatory T cell, IL-10, and TGF-*β* and values of parameters *α*_3_, *γ*_2_, *p*_1_, *p*_2_, *p*_3_, and *p*_4_ are all zeroes. We simulate the optimal controlled model in different scenarios. Firstly, we divide into two cases of the initial value of tumor cells, 10^7^ in Figure 3 and 10^8^ in Figure 4. Secondly, Figures 3 and 4 divided into two cases: the model with or without immunosuppressive effects. The optimality system is solved by using the Runge-kutta fourth-order scheme. The optimal strategy is obtained by solving the state system, the adjoint system, and the transversality conditions. We use Forward-Backward method [18–21] to solve the optimal system.

In our numerical simulation, first we start to solve the state system (1) using the Runge-kutta fourth-order forward in time with a guess for the controls over the simulation time. Then, using the current iteration of the state equations in the system (1), the adjoint equations in the system (10) are solved by a backward method with the transversality conditions (11). We update the controls by using a convex combination of the controls in the previous iterations if the values of unknowns at the previous iteration are very close to the ones at the present iteration. In Figures 3 and 4, (a) represents scenarios for the case when cost for two therapies is the same ratio. (b) represents scenarios for the case when the cost of immunotherapy is more expensive than the cost of chemotherapy. (c) represents scenarios for the case when the cost of chemotherapy is more expensive than the cost of immunotherapy. According to these scenarios, we certify that when the model has immunosuppressive effects, the optimal treatment strategy may change based on treatment cost.

In Figures 3(c) and 4(c), our simulation results show that if the cost of chemotherapy is more expensive than that of immunotherapy, then the optimal treatment strategy for the model without immunosuppressive effects needs to be taken for a longer time for chemotherapy comparing immunotherapy. On the other hand, when the model has immunosuppressive effects and the cost of chemotherapy is more expensive than that of immunotherapy, the optimal treatment strategy for the model needs to be taken for a longer time for immunotherapy comparing chemotherapy. In other words, the optimal treatment strategy may be changed by immunosuppressive effects.

**Figure 5:**
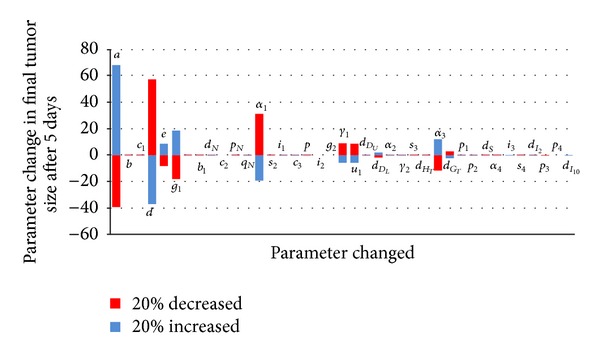
Numerical parameter sensitivity.

In order to find the parameter factors that exert a strong impact on model outcome, we use the numerical parameter sensitivity analysis. For the sensitivity analysis, one parameter value in the model is increased and decreased by 20 percent and the other parameter values are fixed. After 5 days, we plot tumor sizes depending on the model parameters. In Figure 5, the solid red line and blue line represent change rates of tumor cell numbers when a special parameter value was decreased 20 percent and when parameter value was increased 20 percent, respectively. From this we can check that during 5 days, tumor size is highly sensitive to parameters *a* (tumor growth), *d* (strength of immune system), and *α*_1_ (rate of IL-2 and DC induced CD8^+^T cell activation) in order of list.

## 4. Discussion and Conclusions

We constructed a mathematical model describing tumor-immune interactions under immune suppression. From this model, we suggested a treatment protocol for each of chemotherapy and immunotherapy. We investigated the dynamics which effectiveness and efficiency of two therapies by changing of initial tumor cell numbers. We use optimal control techniques and numerical simulation to find a combined therapy strategy for treatment of the tumor. By using Pontryagin's maximum principle, we derived the necessary conditions of optimality for the control system.

To analyse the parameter sensitivity, we plotted the percentage change in tumor size from day zero to day five as a result of changing each of the model parameters by 20% in both directions. From such sensitivity analysis, we found out some special parameters that have a strong influence on tumor growth. Our optimal control experiments demonstrated how chemotherapy and immunotherapy might be combined for more effective treatment. We showed that combined therapy is more effective than each therapy; that is, the number of tumor cells decreases in special parameter sets. In addition, we found out that if the cost of chemotherapy is more expensive than that of immunotherapy, then the optimal treatment strategy for the model without immunosuppressive effects needs to be taken for a longer time for chemotherapy comparing immunotherapy. On the other hand, when the model has immunosuppressive effects and the cost of chemotherapy is more expensive than that of immunotherapy, the optimal treatment strategy for the model needs to be taken for a longer time for immunotherapy comparing chemotherapy.

Even though the methodology of this paper is standard, we provided a key process to develop the optimal control problem related to the cancer model. Based on the parameter sensitive analysis, we formulated an optimal control problem related to the tumor-immune interaction under immune suppression, which is a fresh idea in the optimal control problems. Moreover, this paper gives theoretical and experimental results in the sense of mathematical analysis but if we would have any field data for some cancer treatment and patients, then our results will be applied to the cancer model as well as some other disease models very well.

## Figures and Tables

**Table 1:** Equation terms and parameter descriptions.

State	Term	Parameter	Description
*T*(*t*)	*aT*(1 − *bT*)	*a* = 0.431 (Day^−1^), *b* = 1.02 × 10^−9^ [5]	Tumor growth term
	*c*_1_ *TN*	*c*_1_ = 3.177 × 10^−13^ (Cells^−1^Day^−1^) [7]	NK cells-induced tumor death
	$\frac{dTE}{E_{T}+eT}$	*d* = 0.9 (Day^−1^) *e* = 1.2 [13]	CD8^+^T cells-induced tumor death
	$\frac{1}{\left(1+g_{1}(G_{T}/E_{T}))(1+(S/s_{1})\right)}$	*g*_1_ = 1.2 [13]	Suppression of CD8^+^T cell activity due to TGF-*β* and regulatory T cell
	*k*_*T*_(1 − *e* ^−*M*^)*T*	*k*_*T*_ = 0.9 (Day^−1^) [7]	Chemotherapy-induced tumor death

*N*(*t*)	*b*_1_	*b*_1_ = 3121875 (Day^−1^) [7]	Production of NK cell
	*d*_*N*_ *N*	*d*_*N*_ = 0.0125 (Day^−1^) [7]	Turnover of NK cell
	*c*_2_ *TN*	*c*_2_= 3.177 × 10^−13^ (Cells^−1^Day^−1^) [7]	NK death by exhaustion of tumor killing resources
	$\frac{p_{N}NI_{2}}{q_{N}+I_{2}}$	*p*_*N*_ = 0.0668 (Day^−1^), *q*_*N*_ = 250360 (IU/L) [7]	Stimulatory effect of IL-2 on NK cell
	*k*_*N*_(1 − *e* ^−*M*^)*N*	*k*_*N*_ = 0.6 (Day^−1^) [5]	Chemotherapy-induced NK cell death

*E*_*T*_(*t*)	$\frac{\alpha_{1}I_{2}D_{L}m_{E}}{\left(1+(S/s_{2}))(i_{1}+I_{2})(d_{1}+D_{L}\right)}$	*α*_1_ = 16 (Day^−1^), *s*_2_ = 580000 (IU/L) *i*_1_ = 4909 (IU/L), *d*_1_ = 579579, *m*_*E*_ = 526800 [13]	Proliferation of CD8^+^T cells
	*c*_3_ *E*_*T*_ *T*	*c*_3_ = 3.42 × 10^−10^ (Cells^−1^Day^−1^) [7]	CD8^+^T cell death by exhaustion of tumor killing resources
	*k*_*E*_*T*__(1 − *e* ^−*M*^)*E*_*T*_	*k*_*E*_*T*__ = 0.6 (Day^−1^) [5]	Chemotherapy-induced tumor death

*D*_*U*_(*t*)	$\frac{pT}{\left(1+(I_{10}/i_{2}))(1+(G_{T}/g_{2})\right)}$	*p* = 0.1 (Day^−1^), *i*_2_ = 1200 (IU/L) *g*_2_ = 2 × 10^7^ (cell) [13]	Proliferation of mature unlicensed dendritic cell
	$\frac{\gamma_{1}D_{U}}{1+(D_{U}/m_{H})}$	*γ*_1_ = 0.5 (Day^−1^), *m*_*H*_ = 1053600 [13]	Licensing of dendritic cell upon encounter with CD4^+^T cell
	*d*_*D*_*U*__ *D*_*U*_	*d*_*D*_*U*__ = 0.14 (Day^−1^) [13]	Turnover of CD8^+^T cell
	*k*_*D*_*U*__(1 − *e* ^−*M*^)*D*_*U*_	*k*_*D*_*U*__ = 0.05 (Day^−1^) [5]	Chemotherapy-induced mature unlicensed dendritic cell death

*D*_*L*_(*t*)	*d*_*D*_*L*__ *D*_*L*_	*d*_*D*_*L*__ = 0.5 (Day^−1^) [13]	Turnover of CD8^+^T cell

*H*_*T*_(*t*)	$\frac{\alpha_{2}I_{2}(D_{L}+D_{U})m_{H}}{\left(1+(S/s_{2}))(i_{1}+I_{2})(d_{1}+D_{L}+D_{U}\right)}$	*α*_2_ = 1.9 (Day^−1^) [13]	Proliferation of CD4^+^T cell
	$\frac{\gamma_{2}H_{T}S}{s_{3}+S}$	*γ*_2_ = 0.022 (Day^−1^), *s*_3_ = 34000 (IU/L) [13]	Converting of CD4^+^T cell to regulatory T cell by TGF-*β*
	*d*_*H*_*T*__ *H*_*T*_	*d*_*H*_*T*__ = 0.1 (Day^−1^) [13]	Turnover of CD4^+^T cell
	*k*_*H*_*T*__(1 − *e* ^−*M*^)*H*_*T*_	*k*_*H*_*T*__ = 0.6 [5]	Chemotherapy-induced CD4^+^T cell death

*G*_*T*_(*t*)	$\frac{\alpha_{3}I_{2}D_{L}m_{G}}{\left(i_{1}+I_{2})(d_{1}+D_{L}\right)}$	*α*_3_ = 3.6 (Day^−1^), *m*_*G*_ = 175900 [13]	Proliferation of regulatory T cell
	*d*_*G*_*T*__ *G*_*T*_	*d*_*G*_*T*__ = 0.1 (Day^−1^) [13]	Turnover of regulatory T cell
	*k*_*G*_*T*__(1 − *e* ^−*M*^)*G*_*T*_	*k*_*G*_*T*__ = 0.6 [5]	Chemotherapy-induced regulatory T cell death

*S*(*t*)	*p*_1_ *G*_*T*_	*p*_1_ = 3.6 × 10^−4^ (IU/LCells^−1^Day^−1^) [13]	Production of TGF-*β* by regulatory T cell
	*p*_2_ *T*	*p*_2_ = 2.2 × 10^−3^ (IU/LCells^−1^Day^−1^) [13]	Production of TGF-*β* by tumor cell
	*d*_*S*_ *S*	*d*_*S*_ = 14.3 (Day^−1^) [13]	Turnover of TGF-*β*

*I*_2_(*t*)	$\frac{\alpha_{4}H_{T}}{\left(1+(I_{10}/i_{3}))(1+(S/s_{4})\right)}$	*α*_4_ = 0.278 (IU/LCells^−1^Day^−1^), *i*_3_ = 2250 (IU/L), *s*_4_ = 18000 (IU/L) [13]	Production of IL-2
	*d*_*I*_2__ *I*_2_	*d*_*I*_2__ = 12.5 (Day^−1^) [13]	Turnover of IL-2

*I*_10_(*t*)	*p*_3_ *G*_*T*_	*p*_3_ = 4.2 × 10^−5^ (IU/LCells^−1^Day^−1^) [13]	Production of IL-10 by regulatory T cell
	*p*_4_ *T*	*p*_4_ = 3.9 × 10^−7^ (IU/LCells^−1^Day^−1^) [13]	Production of IL-10 by tumor cell
	*d*_*I*_10__ *I*_10_	*d*_*I*_10__ = 20 (Day^−1^) [13]	Turnover of IL-10

*M*(*t*)	*d*_*M*_ *M*	*d*_*M*_ = 0.9 (Day^−1^) [5]	Excretion and elimination of medicine toxicity
